# Size-Dependent Polymeric Nanoparticle Distribution in a Static versus Dynamic Microfluidic Blood Vessel Model: Implications for Nanoparticle-Based Drug Delivery

**DOI:** 10.1021/acsanm.3c00481

**Published:** 2023-04-24

**Authors:** Sara Gimondi, Helena Ferreira, Rui L. Reis, Nuno M. Neves

**Affiliations:** † 3B’s Research Group, I3Bs−Research Institute on Biomaterials, Biodegradables and Biomimetics, University of Minho, Headquarters of the European Institute of Excellence on Tissue Engineering and Regenerative Medicine, AvePark, Parque de Ciência e Tecnologia, Zona Industrial da Gandra, 4805-017 Barco, Guimarães, Portugal; ‡ ICVS/3B’s−PT Government Associate Laboratory, Braga/Guimarães, Portugal

**Keywords:** microfluidics, nanoparticles, size control, vascular barrier, crossing rate, static model, dynamic model

## Abstract

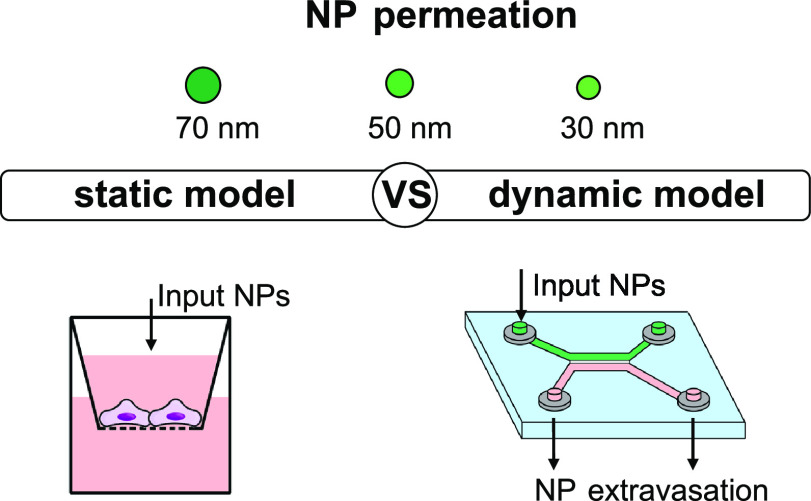

Nanoparticles (NPs) have been widely investigated in the nanomedicine field. One of the main challenges is to accurately predict the NP distribution and fate after administration. Microfluidic platforms acquired huge importance as tools to model the *in vivo* environment. In this study, we leveraged a microfluidic platform to produce FITC-labeled poly(lactide-*co*-glycolide)-*block*-poly(ethylene glycol) (PLGA-PEG) NPs with defined sizes of 30, 50, and 70 nm. The study aimed to compare the ability of NPs with differences of 20 nm in size to cross an endothelial barrier using static (Transwell inserts) and dynamic (microfluidic perfusion device) *in vitro* models. Our results evidence a size-dependent NP crossing in both models (30 > 50 > 70 nm) and highlight the bias deriving from the static model, which does not involve shear stresses. The permeation of each NP size was significantly higher in the static system than in the dynamic model at the earliest stages. However, it gradually decreased to levels comparable with those of the dynamic model. Overall, this work highlights clear differences in NP distribution over time in static versus dynamic conditions and distinct size-dependent patterns. These findings reinforce the need for accurate *in vitro* screening models that allow for more accurate predictions of *in vivo* performance.

## Introduction

Nanoparticles (NPs) are highly used in numerous studies because of their dimensions that translate into unique physical and chemical characteristics.^1−3^ Particularly in nanomedicine, they can overcome the main limitations faced by conventional therapies by improving the pharmacokinetics and pharmacodynamics of a drug.^4,5^ However, NPs should be thoughtfully designed in terms of composition, size, and shape to obtain the desired effects after administration. One of the NPs’ physical properties that highly influences their behavior *in vitro* and *in vivo* is the size.^6,7^ Indeed, NPs’ size plays a crucial role in cellular uptake, circulation time, biodistribution, and clearance.^8^ Upon administration, depending on the delivery route of choice, the entire dose or just a fraction of NPs will end up in the blood circulation. The circulatory system is composed of blood vessels strictly organized along the body, which have the function of transporting nutrients, oxygen, carbon dioxide, and blood cells to different tissues and organs. Consequently, the circulatory system is also important for NPs’ delivery to their target. However, once in the bloodstream, NPs are subjected to hemodynamic parameters, such as blood flow rate, flow disturbances, and wall shear stresses.^9^ These stresses, as well as the interaction with blood cells and vessels, influence the NPs’ distribution and, consequently, their efficacy.^10^ In particular, NPs’ size plays a crucial role in their biodistribution.^11,12^

In the last decades, the importance of *in vitro* models has risen mainly as a result of ethical concerns and the limited predictive value of the human response when animal models are used. In comparison to animal-based assays, *in vitro* studies provide several advantages, such as (i) allowing for rapid screening of several compounds; (ii) enabling real-time monitoring of several parameters, such as pH, metabolism, and cell cytotoxicity; (iii) permitting to recreate pathological conditions to deepen the knowledge of their mechanisms; (iv) allowing for reducing the drug dose needed for the evaluation of its efficacy/toxicity; (v) guaranteeing a high level of standardization; and (vi) ensuring controlled testing conditions.^13,14^ Improvements in this area are obtained mainly by the development of organ-on-a-chip and *in vitro* screening platforms.^15−18^ Particularly, microfluidic cell culture systems are advanced tests to assess NPs’ efficacy and toxicity. Moreover, because of their unique versatility, microfluidic devices can be designed to mimic several biological barriers, addressing, consequently, multiple challenges.^19,20^

Some devices already demonstrated their feasibility to mimic the blood–brain barrier (BBB).^21,22^ Interestingly, these platforms can be exploited to study the NPs’ permeability and accumulation in different physiological^23−25^ and physiopathological^26−28^ conditions. For instance, the integration of shear stress into *in vitro* models enables one to obtain more refined platforms for screening nanomaterials.^29^ Indeed, the shear stress is one key factor influencing NPs’ uptake and overall interaction with cells.^30^ The shear stress is defined as the frictional force generated by a biological fluid against the apical cell membrane, with its value being directly proportional to the fluid velocity.^31^*In vivo*, several adherent cell types are constantly exposed to this force, such as blood vessels, the lymphatic network, and nephrons. This mechanical stimulus influences the adhesion properties and the physiological behavior of cells, which react by ion channel activation, gene expression, and reorganization of the entire cell layer and permeability.^32^ All these events triggered by the shear stress are able to affect the cells’ interaction with NPs.^33^

The possibility of predicting the NPs’ in vivo fate and interactions is crucial to produce a carrier with the desired properties. The most interesting range of NP sizes for intracellular delivery is below 100 nm.^34^ Moreover, research studies reported that even minor variations in size in the subhundred nanometer range can have a substantial impact upon the way NPs interact with cells and other biological structures.^35−37^ However, many conventional strategies for synthesizing NPs lack accurate control over the experimental parameters. This generally leads to the production of NPs with inconsistent or unpredictable sizes. One promising approach to enhance NP synthesis involves microfluidic devices to precisely control the flow and mixing of reactants, leading to highly uniform and monodisperse NPs.^38−40^

In this study, we employed a microfluidic platform that allows for the narrow production of poly(lactide-*co*-glycolide)-*block*-poly(ethylene glycol) (PLGA-PEG) NPs with defined sizes of 30, 50, and 70 nm. Then, the ability of these NPs with 20 nm difference in size to cross an endothelial barrier in dynamic and static conditions was evaluated. To do so, we investigated the most widely used approach of Transwell inserts, which represent a static condition, in comparison with a dynamic system represented by a microfluidic perfusion device. Indeed, the assessment of NP crossing by passive diffusion in static conditions does not consider the effect of blood flow in the transendothelial transport of NPs.^41,42^ The obtained results highlight the importance of using *in vitro* systems that can mimic *in vivo* conditions to obtain valuable data that can be used to predict NP performance in humans.

## Experimental Section

### Materials

PLGA-PEG with PEG and PLGA average molecular weight (MW) of 2 and 11.5 kDa, respectively; PLGA-FITC with average MW of 15 kDa; phosphate-buffered saline (PBS); and fluorescein isothiocyanate-dextran (70 kDa) were purchased from Sigma-Aldrich (PT). Acetone was purchased from Honeywell Riedel-de Haën (FR). Vivaspin 20 MWCO (30 kDa) and dialysis devices (20 kDa) were purchased from Laborspirit (PT). The Quant-IT PicoGreen dsDNA Assay Kit was purchased from Invitrogen (NL). Cell culture inserts for 24-well plates (pore diameter of 1 μm) were purchased from dDBIOLAB (ES). Dulbecco’s modified Eagle medium (DMEM) was purchased from Sigma-Aldrich (PT). Fetal bovine serum (FBS) and antibiotic/antimycotic solution were acquired from Gibco (NL). TrypLE Express (1×) was purchased from Alfagene (PT). Micromixer Chip part #3200401 was purchased from Dolomite (PT). The BE-Doubleflow microfluidic device (height 0.3 mm, width 1.5 mm, length 46 mm, and pore diameter of 1 μm) was purchased from BEOnChip (ES). Water obtained from a Milli-Q Direct Water Purification System was used in all experiments.

### PLGA-PEG/FITC NP Synthesis

**Figure 1:**
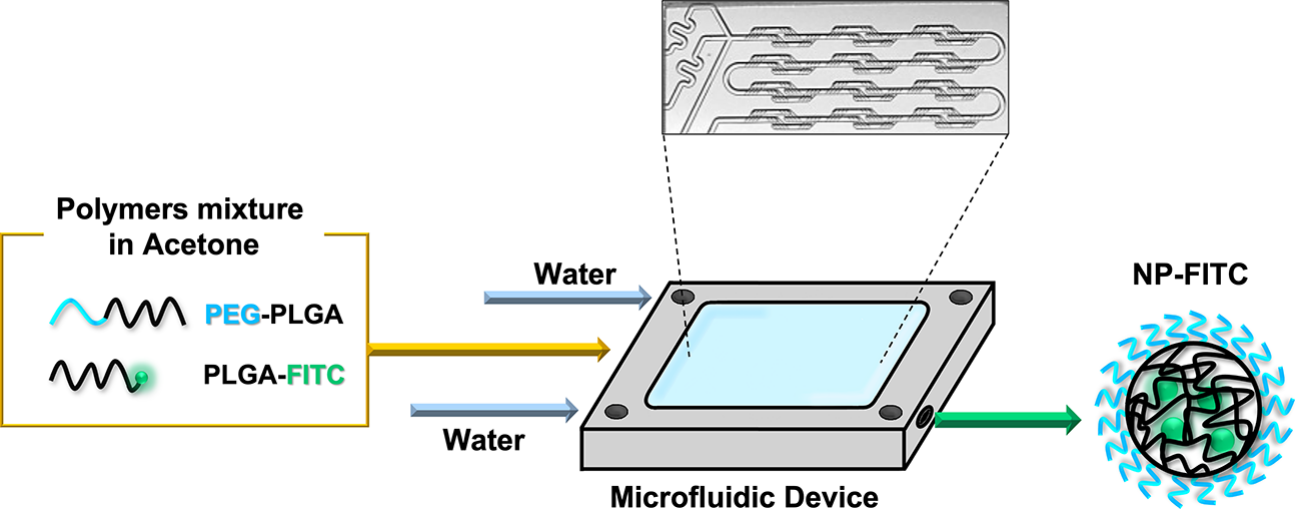
NP synthesis. NP-FITC were synthesized by exploiting a micromixer chip that allowed the rapid mix of water with a solution of PLGA-PEG and PLGA-FITC in acetone. Water flowed in the lateral channels, whereas the polymeric solution in acetone was flowed in the middle channel. The final product is represented by polymeric NPs constituted by hydrophilic PEG on the outside and the hydrophobic portion of PLGA-FITC inside the NPs.

Polymeric NPs were synthesized using a micromixer chip device (Figure 1), as previously described.^43^ It allows for enhancing the mixing time toward the channels, resulting in narrow-sized NPs. The chip was made of glass and presented an internal channel cross section of 125 × 350 μm and 50 × 125 μm (depth × width) and 12 mixing stages. To obtain fluorescently labeled NPs (NP-FITC), PLGA-PEG was mixed with PLGA-FITC in an acetone solution in a ratio of 5:1. The experimental setup consisted of two syringes loaded with water that were controlled simultaneously by a double syringe pump that pushed the liquid through the side channels of the chip. The organic solution was loaded into a syringe controlled by a single pump and feeding the central channel (Figure 1). The synthesis was carried out with a flow rate ratio between the organic phase and aqueous phase of 0.1 and a total flow rate of 550 μL/min. To obtain NPs with different sizes, namely, 30, 50, and 70 nm, and polymer concentrations of 0.3, 2, and 6 mg/mL were used, respectively. At the end of the NP synthesis, acetone was removed by evaporation in a rotary evaporator (RE-301, Stuart; UK). Finally, the NP-FITC with different sizes were stored at 4 °C until further use.

### NP Characterization

#### Size Distribution and Zeta-Potential Determinations

The size and polydispersity index (PDI) of the NPs were evaluated by dynamic light scattering (DLS), and the zeta (ζ)-potential was determined by laser Doppler microelectrophoresis in a Zetasizer Nanoseries ZS equipment (Malvern). The samples were diluted in ultrapure water to obtain a final concentration of 0.25 mg/mL. The measurements were performed at 25 °C in square disposable polystyrene cuvettes or dip cells for DLS or laser Doppler microelectrophoresis, respectively, using a refractive index = 1.330, a dielectric constant = 78.5, and a viscosity (25 °C) = 0.8872 cP. For the stability assay, NP-FITC were diluted in PBS buffer (pH 7.4), and the measurements were performed using the same values of refractive index and viscosity but using a dielectric constant = 79.0.

#### NP Fluorescence

The fluorescence emission (FE) of the NP-FITC was carried out in an FP-8000 Series spectrofluorometer (Jasco). Each sample of NPs was diluted in DMEM (FBS free) at the final concentration of 0.25 mg/mL and read in triplicate. The temperature during all measurements was kept at 25 °C. The excitation wavelength was set at 490 nm, and the spectrum was recorded from 500 to 600 nm. The output of each reading was automatically corrected by blank subtraction (DMEM without FBS and NP-FITC).

#### Stability Studies

To evaluate the stability over time, the NP-FITC suspensions were diluted to 0.25 mg/mL in PBS buffer (pH 7.4). The resulting suspension was transferred to a dialysis bag of 20 kDa and kept at 4 °C under gentle stirring for 1 month. At each time point, aliquots were collected for characterization. The integrity of NPs was also evaluated under shear conditions. NPs resuspended in PBS (0.25 mg/mL) were dispensed inside the microfluidic device at 55 μL/min for 4 h. The device was used without cells to guarantee the free distribution of the NPs within both channels. After the incubation time, NPs were characterized by DLS, laser Doppler microelectrophoresis, and fluorescence spectroscopy as described above.

#### SEM Analysis

The morphology of the NP-FITC was evaluated by scanning electron microscopy (SEM). The NP samples were diluted with ultrapure water at a final concentration of 0.1 mg/mL, and a volume of 0.25 μL was spotted on the top of a mica sample disk (1 drop/sample/mica disk) and left to dry overnight. Afterward, the samples were sputter-coated (EM ACE600, Leica; PT) with a thin layer (8–12 nm) of palladium and analyzed by a high-resolution field emission scanning electron microscope (Auriga Compact, ZEISS). Microphotographs were recorded at 5000×. The same parameters were used to analyze the NPs sedimented on top of the Transwell membrane. Cell-free Transwells were filled on the apical compartment with the NP suspension (0.2 mg/mL), whereas the basolateral compartment was filled with an NP-free medium. After 4 h incubation, the apical solution was aspirated, the membrane of the Transwell was carefully removed by cutting its perimetry with a scalpel, and then SEM images were acquired as previously mentioned.

### Biological Assays

The NP-FITC’s ability to cross a biological layer was assessed on a human umbilical vein cell line (Ea.hy926 cells; ATCC). The EA.hy926 cell line was selected because it is well-established and widely used in the field of endothelial cell biology.^44^ Additionally, it has been shown that it retains many of the physiological features of primary endothelial cells, which makes it a suitable model for investigating vascular function.^45,46^

**Figure 2:**
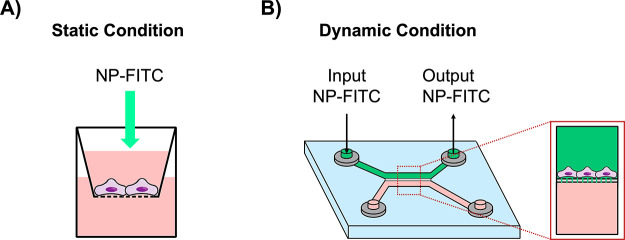
*In vitro* models. Experimental setup of the static (A) and dynamic (B) *in vitro* models.

NPs’ performance was validated in static and dynamic conditions. For the static condition, cell culture inserts for 24-well plates were employed (Figure 2A), whereas for the dynamic system, a versatile microfluidic device composed of two channels separated by a membrane was used (Figure 2B). To allow for the comparison between the models, both culture inserts and microfluidic chip were selected with the same characteristics, namely, material composition (polyethylene terephthalate, PET), pore membrane size (1 μm), and pore density (2 × 10^6^ pore/cm^2^).

#### Cell Seeding in Transwell

To evaluate the NP behavior in the static condition, Ea.hy926 cells cultured in high-glucose DMEM supplemented with FBS and antibiotic/antimycotic solution (10,000 U mL^–1^ penicillin G sodium, 10,000 μg mL^–1^ streptomycin sulfate, and 25 μg mL^–1^ amphotericin B) were seeded at a density of 3.6 × 10^3^ cells/mm^2^ in Transwell inserts for 24-well culture plates. To ensure the formation of a cell layer with maximal cell confluence, the plates were incubated for 3 days at 37 °C in a humidified atmosphere with 5% CO_2_. After this period of time, the medium was gently removed from the apical and basolateral compartments, and cells were washed three times with DMEM without FBS to avoid its interference with the fluorescence measurements. This medium was also used to fill both compartments. Afterward, NP-FITC were added to the apical compartment at the final concentration of 250 μg/mL. The same volume of vehicle (ultrapure water) was used as a control (not treated condition (NT)). At defined time points (1, 2, and 4 h), the liquid from the basolateral compartment was removed. A Transwell without cells but treated with NP-FITC was used as a blank (negative control) to set the threshold of maximum NP-FITC crossing for each time point. Samples were then analyzed by fluorescence spectrometry (FP-8000 Series spectrofluorometer, Jasco).

#### Cell Seeding in the Microfluidic Device

The NP crossing in dynamic conditions was evaluated by employing a microfluidic device for cell culture, as previously indicated (Figure 2B). The microfluidic device BE-Doubleflow is a versatile tool for cell culture under biomimetic conditions. This chip, made of biocompatible plastic, is gas-impermeable and presents excellent optical properties with high transparency and low autofluorescence. The design of the BE-Doubleflow device consists of two channels connected by a porous membrane of 1 μm pore size. Its design allows for flowing the solutions in both channels, and with the help of a screw-like inlet and outlet, it is possible to connect it through tubes to a syringe pump system. The device also has evaporation reservoirs that can be filled with PBS or water to prevent medium evaporation if it is open. Moreover, the internal channels have a hydrophilic coating that enables their filling with aqueous solutions and promotes cell adhesion.

To obtain a monolayer inside the upper channel, Ea.hy926 cells were seeded at the density of 1.5 × 10^3^ cells/mm^2^ by flowing 100 μL of their suspension in DMEM supplemented with FBS and antibiotic/antimycotic solution (10,000 U mL^–1^ penicillin G sodium, 10,000 μg mL^–1^ streptomycin sulfate, and 25 μg mL^–1^ amphotericin B) through the inlet. Then, the cells were cultured for 3 days at 37 °C in a humidified atmosphere with 5% CO_2_. Once the layer formation was ensured, the apical and basal channels of the device were washed very carefully with DMEM FBS-free. Then, the chip was sealed with proper tubing and sleeves that allowed for connecting the device to the syringe pump system. In the syringe pump, a 5 mL syringe containing NP-FITC suspension in DMEM FBS-free at the concentration of 250 μg/mL was placed. A solution of DMEM FBS-free and the vehicle was used for the NT condition. Finally, the syringe pump was set up to dispense the NP suspension in the medium for 4 h at a flow rate of 55 μL/min (shear stress 0.4 dyn/cm^2^) to reproduce the physiological shear stress.^47^ After each time point (1, 2, and 4 h), the liquid from the basolateral compartment was collected. For each type of NP-FITC tested, a device with no cultured cells on it was used as a blank (negative control) to set the threshold of maximum NP-FITC crossing for each time point.

#### Permeability Assays

Ea.hy926 cells were cultured in Transwell inserts or in the microfluidic device BE-Doubleflow as described above. The basolateral compartment was filled with DMEM FBS-free, whereas the apical compartment was replaced with DMEM FBS-free containing 2 mg/mL 70 kDa FITC-dextran. The amount of FITC-dextran that crossed the monolayer was measured in the medium of the basolateral compartment after 4 h incubation at 37 °C and 5% CO_2_ by fluorescence spectrometry at 485 nm excitation and 530 nm emission wavelengths.

#### TEER Measurement

The transendothelial electrical resistance (TEER) was measured after 3 days of culture using an electrical resistance system (Millicell ERS-2). The cells were incubated with the different NP-FITC formulations or the vehicle for 4 h, and then the electrodes were dipped in the apical and basolateral compartment of the Transwell. To calculate the TEER (Ω × cm^2^), we used the formula below:
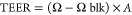
where Ω is the resistance of a treatment condition, Ω_blk_ is the resistance of the blank condition (no cells), and *A* is the surface area of the monolayer.

#### DNA Quantification

The Quant-IT PicoGreen dsDNA Assay Kit was used according to the manufacturer’s instructions. Briefly, cells seeded and treated as described above were gently detached by the addition of TrypLE Express after 4 h incubation. Afterward, the cell suspension was collected and centrifuged at 300*g* for 5 min, and the supernatant was removed. Ultrapure water (500 μL) was added to lyse the cells and frozen at −80 °C until further analysis. Before DNA quantification, the samples were thawed and sonicated for 15 min. DNA standards were prepared at concentrations ranging from 0 to 1 μg/mL in ultrapure water. The fluorescence was measured using a microplate reader (Synergie HT, Bio-Tek) at excitation and emission wavelengths of 485 nm and 528 nm, respectively. The DNA concentration of the samples was obtained from the standard curve and expressed as a percentage against the NT.

#### NP Crossing Rate Quantification

To quantify the amount of NP-FITC that was able to cross the endothelial layer in static and dynamic conditions, we analyzed the basal medium after 1, 2, and 4 h incubation by fluorescence spectrometry (FP-8000 Series spectrofluorometer, Jasco), as previously described. The determination of the different NPs’ crossing rate was calculated using the following formula:
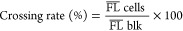
where  is the average fluorescence of the basolateral medium collected after cell incubation with the NP-FITC, whereas  is the average fluorescence of the basolateral medium determined after incubating the NP-FITC in Transwells without cells (blank, blk).  was calculated for each time point and NP formulation. The same formula was applied to calculate the crossing rate for each NP-FITC at different time points in the dynamic model.

### Statistical Analysis

Experimental outcomes are expressed as mean ± standard deviation (SD) of three independent experiments. Two-way ANOVA and multiple comparison were used to determine significant differences between means. *P* < 0.05 was considered statistically significant.

## Results and Discussion

### Microfluidic Synthesis and Characterization of NP-FITC

NP-FITC with sizes of 30, 50, and 70 nm were synthesized using a microfluidic device with a specific design of the internal channels that allows for enhancing the mixing of three fluid streams^48−50^ (Figure 1). The microfluidic device employed is a lamination-based compact micromixer chip that allows for a controlled nanoprecipitation process.^51−53^ For the NP synthesis, a mixture of PLGA functionalized with one of two different molecules, namely, PLGA-PEG and PLGA-FITC, was used. Therefore, the produced NPs presented an external hydrophilic layer composed of PEG, which shields NPs from aggregation and opsonization. Additionally, the covalent binding of FITC to PLGA guarantees the permanence of the fluorophore into the NPs, avoiding its release over time.^54^ After synthesis, the resulting NPs were characterized in terms of FE, size, PDI, and ζ-potential. Additional information regarding NPs’ size distribution by intensity, volume, and number is reported in the Supporting Information (SI) as Figure S1 and Table S1. They show that a narrow size distribution was obtained for all formulations. As shown in Table 1, the hydrodynamic diameter measures the peak intensity of NP-FITC, resulting in sizes close to 30, 50, and 70 nm. An overall PDI < 0.2 was also obtained (Table 1), demonstrating the NPs’ monodispersity. By enabling the determination of NPs’ hydrodynamic radius in an aqueous environment, DLS gives more representative data of the NPs’ size in biological fluids. Also, NPs presented a negative surface charge due to the nature of the polymer employed^55,56^ (Table 1). The observed differences in zeta-potential values (ranging from −10 to −25 mV) can be related to the different amounts of polymer forming the NPs. Thus, we hypothesize that larger NPs require more polymer during the nucleation process than smaller ones, resulting in slightly different surface charges between sizes.^57^ Although all physicochemical properties of NPs are crucial in determining NPs’ interaction with cells, we believe that the size plays a more important role than surface charge in the current study. Conversely, if the difference in surface charge were more pronounced (e.g., positively charged NPs versus negatively charged NPs), it would likely have a significant impact on the interaction with cells.^58−60^

**Table 1:** NP Characteristics^a^

NP-FITC	intensity (nm)	PDI	ζ-potential (mV)	FE
30 nm	33.8	0.1	–10.9	2850.3
50 nm	52.7	0.1	–16.5	3488.1
70 nm	70.2	0.1	–25.1	4126.3

a NP size, PDI, ζ-potential, and FE at 518 nm. Values are reported as the average value of three independent measurements.

Finally, the FE of all nanoformulations was in the same order of magnitude but presented an increasing trend with the NP-FITC size (30 < 50 < 70 nm; Table 1).

**Figure 3:**
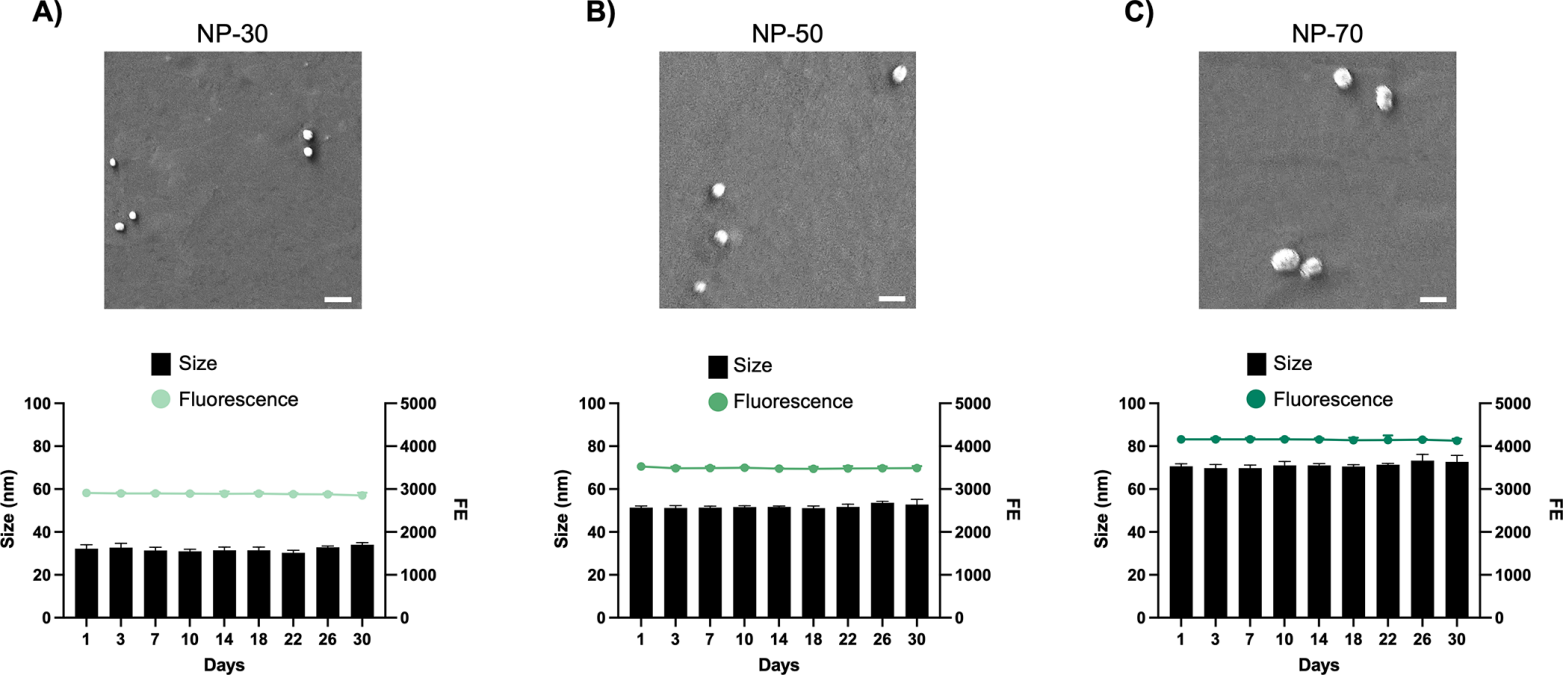
NP-FITC characterization. The obtained NPs of 30 nm (A), 50 nm (B), and 70 nm (C) were evaluated in terms of size (nm) and FE over 30 days. SEM images illustrate the NP morphology (scale bar of 100 nm).

NP-FITC stability in static conditions along 30 days of incubation in PBS at 4 °C is presented in Figure 3. Additionally, the stability of the nanoformulations under shear stress was assessed to ensure NPs’ integrity during the perfusion experiments. The results are reported in Table S2 (SI). The results from the stability tests in both static and dynamic conditions demonstrated that all nanoformulations did not present significant changes in their features during the investigated period of time. Finally, with the NP shape being an important parameter in determining NP circulation time^61^ and interaction with cells,^62^ SEM analysis was performed. The obtained images show that NPs have a spherical shape (Figure 3).

### Cellular Layer Assessment

To assess the integrity of the endothelial monolayer, several indicators, including TEER, permeability, DNA content, and morphology, were examined.

**Figure 4:**
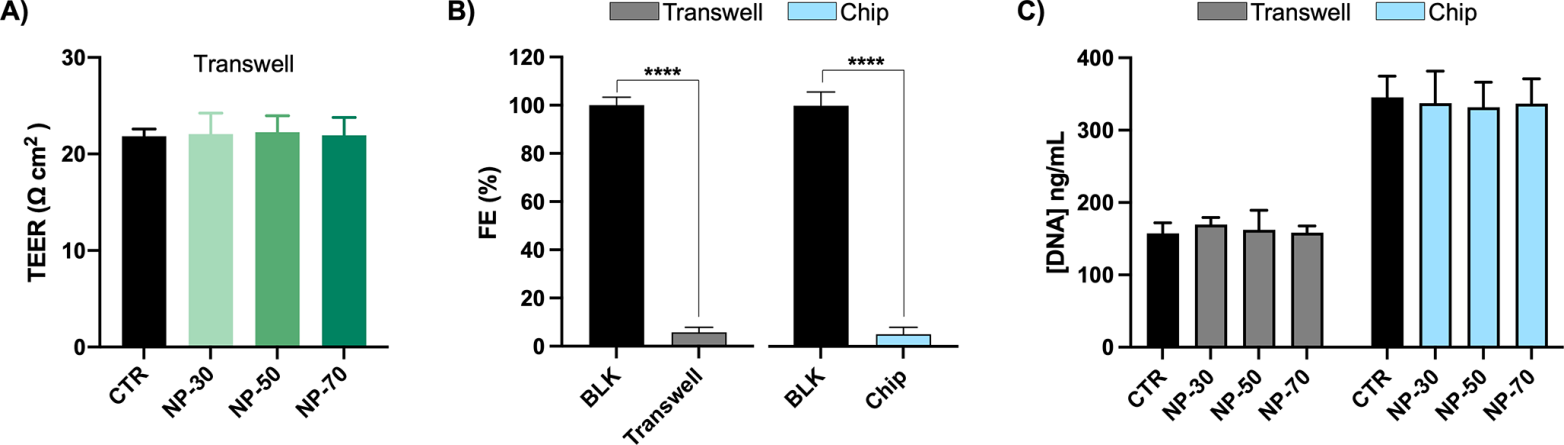
Monolayer integrity assessment. The endothelial monolayer was assessed by TEER measurements (A), FITC-dextran permeability assay (B), and DNA quantification (C). Asterisks (*) denote significant differences: **** = *p* < 0.0001.

The measurement of TEER was only possible on Transwell inserts. The resulting values of TEER are shown in Figure 4A. None of the NP-FITC formulations tested affected the TEER of the endothelial cell layer. Moreover, values around 22 Ω cm^2^ indicate cell confluence in the Transwell inserts, as reported in other works.^63−65^ The FITC-dextran leakage (Figure 4B) was below ≈10% for both models, suggesting good integrity of the monolayer.^66^ Additionally, we ensured that the treatment with NP-FITC did not affect cell viability, which might cause an alteration of the monolayer integrity. For that, we quantified the DNA content of each condition (static and dynamic models) after 4 h of cell incubation. The obtained results confirmed that the treatment with NP-FITC is not cytotoxic and provided insights about the monolayer’s integrity. Indeed, cells were seeded based on the surface area of each model. Thus, a correlation can be obtained between the DNA content of the monolayer seeded on the Transwell and the microfluidic channel. The results showed that the DNA content of the cell layer seeded on the microfluidic device was ≈2× the amount of DNA present on the Transwell, which is consistent with their area: 69 and 33.6 mm^2^, respectively.

Finally, we acquired bright-field images of the monolayer formed inside the microfluidic channel. Human vascular endothelial cells spontaneously organize to form a uniform layer, and when the confluency is reached, they form a cobblestone-like monolayer.^67^ An identical cell arrangement was observed in the microfluidic channel, as illustrated in Figure S2 (SI).

### NP Crossing Rate

When NPs are administered via the intravascular route, endothelial cells represent the first cells with which they need to interact to reach the target tissues. The majority of the studies utilize static cell culture conditions to assess the cellular interaction with NPs. Indeed, relatively few reports about NP–endothelial cell interactions under flow conditions are available. However, they can provide important information to estimate the cellular responses in physiological conditions. Indeed, NP extravasation and circulation time are important parameters to consider if a nanocarrier is designed for intravascular delivery.^68^ After intravascular administration, NP circulation time decreases rapidly after the first hour, reaching a plateau after 4 h.^69^ This is due to NP clearance performed by the liver, spleen, and kidney, which filter the bloodstream. For this reason, early time points of 1, 2, and 4 h were selected. Indeed, Dal et al.^70^ investigated the circulation time of NPs with different sizes in zebrafish embryos, and the results showed that the percentage of NPs in circulation critically dropped after 4 h. A similar study was carried out in mice, showing similar outcomes.^71^ In fact, 24 h after intravenous injection (tail vein), less than 20% of the NPs were detected in the bloodstream. These results show the tendency of NPs to be removed from the blood circulation quickly to then reach a plateau (≈4 h).

**Figure 5:**
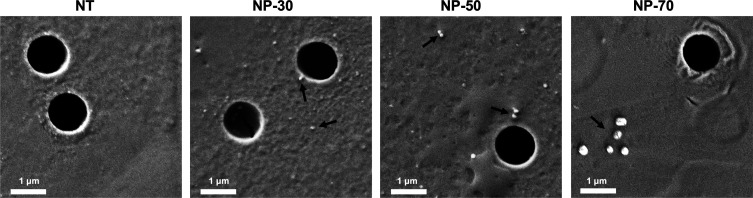
NP sedimentation. SEM images illustrate the Transwell membrane after treatment with vehicle (NT) or 30, 50, and 70 nm NP-FITC in the absence of a cell layer. The arrows indicate some of the NPs sedimented on top of the membrane. Scale bare 1 μm.

Here, we evaluated to which extent the NP-FITC ability to cross biological barriers is affected by static or dynamic environments. For that, we used, respectively, a Transwell culture insert and a microfluidic device, which can more closely mimic the *in vivo* scenario as compared with the static model. At first, we established the maximal amount of each NP-FITC size that was able to pass through the membrane in the static model. We set the 100% threshold based on the fluorescence value obtained by incubating each nanoformulation on a Transwell insert with no cells seeded. This allowed for setting a threshold representing the maximal crossing through the membrane, taking into consideration (i) the NP-FITC that might lie on the membrane surface and (ii) the NP-FITC that can get stuck in the pores as illustrated in Figure 5.

**Figure 6:**
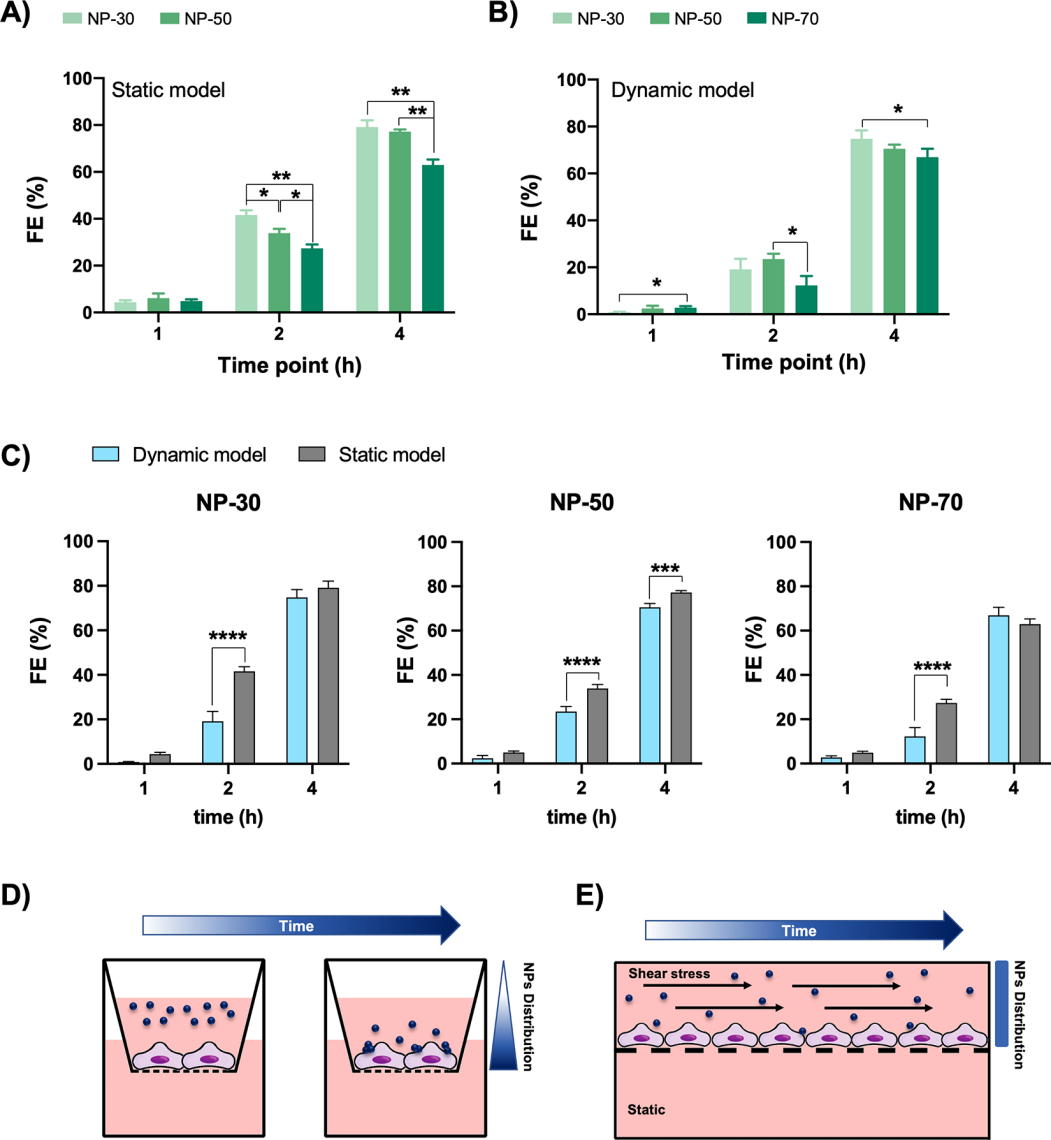
NP-FITC crossing rate. Fluorescence values expressed in percentage (%) of 30, 50, and 70 nm NP-FITC in static (A) and dynamic (B) conditions. Crossing rate comparison between *in vitro* models (C). Scheme illustrating NP-FITC distribution in static (D) and dynamic (E) conditions. Asterisks denote significant difference: **** = *p* < 0.0001, *** = *p* < 0.001, ** = *p* < 0.01, and * = *p* < 0.05.

Once 100% for each NP-FITC size and time point was settled, we assessed their ability to pass through the membrane when a cell monolayer, mimicking a vascular endothelium, is seeded on top of it. Figure 6 shows that all NP-FITC formulations were able to cross the monolayer in a time-dependent rate. In static conditions (Figure 6A), during the first hour of incubation, the measured fluorescence reached around 10% for all NP-FITC with no significant differences. After 2 h, NP-30 reached a 41 ± 2.3% crossing rate followed by NP-50 (34 ± 1.6%) and NP-70 (29 ± 2.5%). After 4 h incubation, NP-30 doubled their permeation, showing the same crossing rate as NP-50 (79 ± 8.3 and 76 ± 2.2%, respectively), whereas NP-70 reached 60 ± 5.0%. This first experiment, in static conditions, showed the size-dependent behavior of the NPs during the interaction with a cell layer, especially after 2 h incubation. At 4 h incubation, the size dependency was reduced, and NP-30 and NP-50 exhibited the same ability to cross the cellular barrier. These results are corroborated by our recent study about the cellular uptake of NPs. Results reported the same size dependency in internalization rate for the same cell line (EA.hy926) and NP formulations.^37^ Indeed, we observed that NP-FITC undergo quick internalization in endothelial cells (30 > 50 > 70 nm). Hence, similar events should be observed in NPs crossing through the endothelial barrier.

A size-dependent permeability was also reported by Brown et al. who investigated the effect of different NP parameters over their penetration across the BBB.^42^ The results showed that the apparent permeability coefficient (*P*_app_) was higher for 50 nm spherical polystyrene NPs compared to larger ones (100 and 200 nm). Additionally, within the same size, spherical protein-based NPs showed a higher permeability across the cellular layer compared to polystyrene NPs, liposomes, and rod-shaped NPs. Thus, the rod shape could reduce the NP association with the endothelium compared with the spherical shape.^21^ Indeed, several studies highlight the importance of NP physicochemical properties in determining their interaction with cells.^72,73^

Despite the mechanism of NP transcytosis being still unclear, NPs tend to be mainly internalized by clathrin-mediated endocytosis (CME), caveolin-mediated endocytosis (CVME), or micropinocytosis.^74^ Subsequently, the cargo of NPs is translocated through the cytoplasm and released by an exocytosis mechanism.^75^ In addition, the endothelium barrier has specialized cell-to-cell junctions that allow for fine regulation of molecules and cellular transversal between the bloodstream and surrounding tissue via the paracellular route.^76^

Although the specific pathways were not investigated in this study, we hypothesize that NP crossing of the cell layer should be driven by the combination of the paracellular and transcellular routes.

The same experiment was replicated in dynamic conditions, and the results are illustrated in Figure 6B. Cells were exposed to a continuous flow of NP-FITC, and after 1 h incubation, NP-50 and NP-70 showed the highest crossing rate values (2.0 ± 1.2 and 2.3 ± 1.3%, respectively), whereas NP-30 barely reached 1.0 ± 0.3%. After 2 h, the NP-30 crossing rate increased by 20× (21 ± 7.0%), reaching the same average rate as NP-50 (24 ± 2.1%). Conversely, NP-70 exhibited a lower crossing rate (16 ± 6.8%). After 4 h, all the NP-FITC formulations demonstrated a similar ability to cross the cell layer (NP-30, 74 ± 3.1%; NP-50, 70 ± 2.2%; and NP-70, 65 ± 3.7%).

In the human body, the physiological wall shear stress ranges between 0.5 and 120 dyn/cm^2^, depending on the vessel type.^77^ From the literature, it is clear that the effects of shear stress on the NPs’ uptake are highly correlated to their features and the experimental settings used.^78,79^ A study performed with different shear stress levels reported a higher uptake of quantum dots for a lower shear stress (0.5 dyn/cm^2^) compared to a higher shear stress (5 dyn/cm^2^).^80^ The NPs’ uptake has been associated with endothelial cytoskeleton reorganization and the formation of membrane ruffles induced by the applied shear stress.^81−83^ Moreover, static *in vitro* studies underestimate the NPs’ uptake and flow interaction compared to *in vivo* assays.^84,85^ A comparison between the NPs’ behavior in the two *in vitro* models is illustrated in Figure 6C. The data obtained from both models indicate that for the static model, the crossing rate is overall higher for all NP-FITC at earlier time points, whereas it tends to decrease to levels observed in dynamic conditions after 4 h for NP-30 and NP-70.

The different outcomes can be related to the stativity or dynamism of the system itself. In fact, as illustrated in Figure 6D,E, when cells are exposed to NP-FITC within a static environment, the NP-FITC tend to accumulate over time on top of the cellular layer due to the gravitational force, as also reported in other studies.^86−88^ This translates into a progressive deposition of NPs over time, increasing the chance of endothelial barrier crossing. Conversely, the use of the BE-Doubleflow device enabled precise control of fluid flow rates, which ensured that cells were subjected to a homogeneous exposure over time of NP-FITC. Indeed, microfluidic chips can improve the statistical efficiency of experiments by increasing consistency and experimental reproducibility.^89,90^

## Conclusions

In this study, we efficiently produced and characterized different NP-FITC formulations by exploiting a microfluidic device that allowed for the precise control of the resulting NP size. The 30, 50, and 70 nm NP-FITC presented a narrow size distribution, good fluorescence signal, negative surface charge, and stability during the experimental period considered. The static and dynamic systems allowed for analyzing the differences in NP distribution in the presence or absence of shear stress, which is one of the main parameters that affect NPs’ interaction with cells and consequent barrier-crossing ability. The results obtained with both models showed a size-dependent behavior, with the highest crossing rate percentage being obtained for the smaller NP-FITC (30 > 50 > 70 nm). The comparison between the two *in vitro* models showed a higher cross rate during the early time points for the static condition compared to the dynamic one. This behavior is strongly related to the shear stress and the distinct NP-FITC distribution in the environment around cells, which influence their ability to cross the endothelial barrier. Thus, our investigation reinforces the need for physiologically accurate *in vitro* models resembling the endothelial barrier to predict *in vivo* behavior. Further advances in this area will aid to reduce the expensive animal studies and related ethical concerns.

## Supporting Information Available

Additional NP characterization (size distribution by intensity, volume, and number), NP stability in dynamic conditions, and dynamic model images (PDF)

## Abbreviations

BBB: blood–brain barrier
Blk: blank
DLS: dynamic light scattering
FE: fluorescence emission
FITC: fluorescein isothiocyanate
NP: nanoparticle
NT: not treated
PET: polyethylene terephthalate
PLGA-PEG: poly(lactide-*co*-glycolide)-*block*-poly(ethylene glycol)
TEER: transendothelial electrical resistance

## References

[ref1] Mitchell M. J.; Billingsley M. M.; Haley R. M.; Wechsler M. E.; Peppas N. A.; Langer R. Engineering Precision Nanoparticles for Drug Delivery. Nat. Rev. Drug Discovery 2021, 20, 101–124. 10.1038/s41573-020-0090-8. 33277608 PMC7717100

[ref2] Mitchell S.; Qin R.; Zheng N.; Pérez-Ramírez J. Nanoscale Engineering of Catalytic Materials for Sustainable Technologies. Nat. Nanotechnol. 2021, 16, 129–139. 10.1038/s41565-020-00799-8. 33230317

[ref3] Violatto M. B.; Casarin E.; Talamini L.; Russo L.; Baldan S.; Tondello C.; Messmer M.; Hintermann E.; Rossi A.; Passoni A.; Bagnati R.; Biffi S.; Toffanin C.; Gimondi S.; Fumagalli S.; De Simoni M.-G.; Barisani D.; Salmona M.; Christen U.; Invernizzi P.; Bigini P.; Morpurgo M. Dexamethasone Conjugation to Biodegradable Avidin-Nucleic-Acid-Nano-Assemblies Promotes Selective Liver Targeting and Improves Therapeutic Efficacy in an Autoimmune Hepatitis Murine Model. ACS Nano 2019, 13, 4410–4423. 10.1021/acsnano.8b09655. 30883091

[ref4] Lima A. C.; Ferreira H.; Reis R. L.; Neves N. M. Biodegradable Polymers: An Update on Drug Delivery in Bone and Cartilage Diseases. Expert Opin. Drug Delivery 2019, 16, 795–813. 10.1080/17425247.2019.1635117. 31220958

[ref5] Morelli L.; Gimondi S.; Sevieri M.; Salvioni L.; Guizzetti M.; Colzani B.; Palugan L.; Foppoli A.; Talamini L.; Morosi L.; Zucchetti M.; Violatto M. B.; Russo L.; Salmona M.; Prosperi D.; Colombo M.; Bigini P. Monitoring the Fate of Orally Administered PLGA Nanoformulation for Local Delivery of Therapeutic Drugs. Pharmaceutics 2019, 11, 658 10.3390/pharmaceutics11120658. 31817781 PMC6955864

[ref6] Wang X.; Liu Y.; Shen C.; Shen B.; Zhong R.; Yuan H. Effect of Particle Size on in Vitro and in Vivo Behavior of Astilbin Nanosuspensions. J. Drug Delivery Sci. Technol. 2019, 52, 778–783. 10.1016/j.jddst.2019.05.005.

[ref7] Jain A. K.; Thareja S. In Vitro and in Vivo Characterization of Pharmaceutical Nanocarriers Used for Drug Delivery. Artif. Cells, Nanomed., Biotechnol. 2019, 47, 524–539. 10.1080/21691401.2018.1561457. 30784319

[ref8] Barar J. Bioimpacts of Nanoparticle Size: Why It Matters?. BioImpacts 2015, 5, 113–115. 10.15171/bi.2015.23. 26457247 PMC4597157

[ref9] Gomez-Garcia M. J.; Doiron A. L.; Steele R. R. M.; Labouta H. I.; Vafadar B.; Shepherd R. D.; Gates I. D.; Cramb D. T.; Childs S. J.; Rinker K. D. Nanoparticle Localization in Blood Vessels: Dependence on Fluid Shear Stress, Flow Disturbances, and Flow-Induced Changes in Endothelial Physiology. Nanoscale 2018, 10, 15249–15261. 10.1039/C8NR03440K. 30066709

[ref10] Fullstone G.; Wood J.; Holcombe M.; Battaglia G. Modelling the Transport of Nanoparticles under Blood Flow Using an Agent-Based Approach. Sci. Rep. 2015, 5, 10649 10.1038/srep10649. 26058969 PMC4462051

[ref11] Sun X.; Rossin R.; Turner J. L.; Becker M. L.; Joralemon M. J.; Welch M. J.; Wooley K. L. An Assessment of the Effects of Shell Cross-Linked Nanoparticle Size, Core Composition, and Surface PEGylation on in Vivo Biodistribution. Biomacromolecules 2005, 6, 2541–2554. 10.1021/bm050260e. 16153091 PMC2533516

[ref12] Jasinski D. L.; Li H.; Guo P. The Effect of Size and Shape of RNA Nanoparticles on Biodistribution. Mol. Ther. 2018, 26, 784–792. 10.1016/j.ymthe.2017.12.018. 29402549 PMC5910665

[ref13] Heymans M.; Sevin E.; Gosselet F.; Lundquist S.; Culot M. Mimicking Brain Tissue Binding in an in Vitro Model of the Blood-Brain Barrier Illustrates Differences between in Vitro and in Vivo Methods for Assessing the Rate of Brain Penetration. Eur. J. Pharm. Biopharm. 2018, 127, 453–461. 10.1016/j.ejpb.2018.03.007. 29602020

[ref14] Bouhaddou M.; Yu L. J.; Lunardi S.; Stamatelos S. K.; Mack F.; Gallo J. M.; Birtwistle M. R.; Walz A. Predicting In Vivo Efficacy from In Vitro Data: Quantitative Systems Pharmacology Modeling for an Epigenetic Modifier Drug in Cancer. Clin. Transl. Sci. 2020, 13, 419–429. 10.1111/cts.12727. 31729169 PMC7070804

[ref15] Guimarães C. F.; Gasperini L.; Ribeiro R. S.; Carvalho A. F.; Marques A. P.; Reis R. L. High-Throughput Fabrication of Cell-Laden 3D Biomaterial Gradients. Mater. Horiz. 2020, 7, 2414–2421. 10.1039/D0MH00818D.

[ref16] Soto F.; Guimarães C. F.; Reis R. L.; Franco W.; Rizvi I.; Demirci U. Emerging Biofabrication Approaches for Gastrointestinal Organoids towards Patient Specific Cancer Models. Cancer Lett. 2021, 504, 116–124. 10.1016/j.canlet.2021.01.023. 33577978

[ref17] Zhou Q.; Castañeda Ocampo O.; Guimarães C. F.; Kühn P. T.; van Kooten T. G.; van Rijn P. Screening Platform for Cell Contact Guidance Based on Inorganic Biomaterial Micro/Nanotopographical Gradients. ACS Appl. Mater. Interfaces 2017, 9, 31433–31445. 10.1021/acsami.7b08237. 28825457 PMC5609122

[ref18] Zhou Q.; Ge L.; Guimarães C. F.; Kühn P. T.; Yang L.; van Rijn P. Development of a Novel Orthogonal Double Gradient for High-Throughput Screening of Mesenchymal Stem Cells–Materials Interaction. Adv. Mater. Interfaces 2018, 5, 1800504 10.1002/admi.201800504.

[ref19] Terrell-Hall T. B.; Ammer A. G.; Griffith J. I. G.; Lockman P. R. Permeability across a Novel Microfluidic Blood-Tumor Barrier Model. Fluids Barriers CNS 2017, 14, 3 10.1186/s12987-017-0050-9. 28114946 PMC5260004

[ref20] Wang H.-F.; Ran R.; Liu Y.; Hui Y.; Zeng B.; Chen D.; Weitz D. A.; Zhao C.-X. Tumor-Vasculature-on-a-Chip for Investigating Nanoparticle Extravasation and Tumor Accumulation. ACS Nano 2018, 12, 11600–11609. 10.1021/acsnano.8b06846. 30380832

[ref21] Nowak M.; Brown T. D.; Graham A.; Helgeson M. E.; Mitragotri S. Size, Shape, and Flexibility Influence Nanoparticle Transport across Brain Endothelium under Flow. Bioeng. Transl. Med. 2020, 5, e10153 10.1002/btm2.10153. 32440560 PMC7237148

[ref22] Hajal C.; Offeddu G. S.; Shin Y.; Zhang S.; Morozova O.; Hickman D.; Knutson C. G.; Kamm R. D. Engineered Human Blood–Brain Barrier Microfluidic Model for Vascular Permeability Analyses. Nat. Protoc. 2022, 17, 95–128. 10.1038/s41596-021-00635-w. 34997242

[ref23] Straehla J. P.; Hajal C.; Safford H. C.; Offeddu G. S.; Boehnke N.; Dacoba T. G.; Wyckoff J.; Kamm R. D.; Hammond P. T. A Predictive Microfluidic Model of Human Glioblastoma to Assess Trafficking of Blood–Brain Barrier-Penetrant Nanoparticles. Proc. Natl. Acad. Sci. U. S. A. 2022, 119, e2118697119 10.1073/pnas.2118697119. 35648828 PMC9191661

[ref24] Ahn S. I.; Sei Y. J.; Park H.-J.; Kim J.; Ryu Y.; Choi J. J.; Sung H.-J.; MacDonald T. J.; Levey A. I.; Kim Y. Microengineered Human Blood–Brain Barrier Platform for Understanding Nanoparticle Transport Mechanisms. Nat. Commun. 2020, 11, 175 10.1038/s41467-019-13896-7. 31924752 PMC6954233

[ref25] Charwat V.; Olmos Calvo I.; Rothbauer M.; Kratz S. R. A.; Jungreuthmayer C.; Zanghellini J.; Grillari J.; Ertl P. Combinatorial in Vitro and in Silico Approach To Describe Shear-Force Dependent Uptake of Nanoparticles in Microfluidic Vascular Models. Anal. Chem. 2018, 90, 3651–3655. 10.1021/acs.analchem.7b04788. 29478320

[ref26] Neto E.; Monteiro A. C.; Leite Pereira C.; Simões M.; Conde J. P.; Chu V.; Sarmento B.; Lamghari M. Micropathological Chip Modeling the Neurovascular Unit Response to Inflammatory Bone Condition. Adv. Healthcare Mater. 2022, 11, 2102305 10.1002/adhm.202102305. PMC11468530 35158409

[ref27] Abdulla A.; Zhang T.; Li S.; Guo W.; Warden A. R.; Xin Y.; Maboyi N.; Lou J.; Xie H.; Ding X. Integrated Microfluidic Single-Cell Immunoblotting Chip Enables High-Throughput Isolation, Enrichment and Direct Protein Analysis of Circulating Tumor Cells. Microsyst. Nanoeng. 2022, 8, 13 10.1038/s41378-021-00342-2. 35136652 PMC8807661

[ref28] Elberskirch L.; Knoll T.; Königsmark R.; Renner J.; Wilhelm N.; von Briesen H.; Wagner S. Microfluidic 3D Intestine Tumor Spheroid Model for Efficient in Vitro Investigation of Nanoparticular Formulations. J. Drug Delivery Sci. Technol. 2021, 63, 102496 10.1016/j.jddst.2021.102496.

[ref29] Kim D.; Lin Y. S.; Haynes C. L. On-Chip Evaluation of Shear Stress Effect on Cytotoxicity of Mesoporous Silica Nanoparticles. Anal. Chem. 2011, 83, 8377–8382. 10.1021/ac202115a. 22032307 PMC3220276

[ref30] Shurbaji S.; Anlar G. G.; Hussein E. A.; Elzatahry A.; Yalcin H. C. Effect of Flow-Induced Shear Stress in Nanomaterial Uptake by Cells: Focus on Targeted Anti-Cancer Therapy. Cancers 2020, 12, 1916 10.3390/cancers12071916. 32708521 PMC7409087

[ref31] Lehoux S. Chapter 2 - Molecular Mechanisms of the Vascular Responses to Hemodynamic Forces. In Biomechanics of Coronary Atherosclerotic Plaque; Ohayon J., Finet G., Pettigrew R. I., Eds.; Biomechanics of Living Organs; Academic Press, 2021; Vol. 4, pp. 49–83. 10.1016/B978-0-12-817195-0.00002-0.

[ref32] White C. R.; Frangos J. A. The Shear Stress of It All: The Cell Membrane and Mechanochemical Transduction. Philos. Trans. R. Soc., B 2007, 362, 1459–1467. 10.1098/rstb.2007.2128. PMC2440408 17569643

[ref33] Nikravesh N.; Diener L.; Chortarea S.; Rippl A.; Wick P. Endothelium Response to Iron Sucrose Nanoparticles in Static Versus Dynamic Culture Model; preprint; In Review, 2021. 10.21203/rs.3.rs-279067/v1.

[ref34] Joudeh N.; Linke D. Nanoparticle Classification, Physicochemical Properties, Characterization, and Applications: A Comprehensive Review for Biologists. J. Nanobiotechnol. 2022, 20, 262 10.1186/s12951-022-01477-8. PMC9171489 35672712

[ref35] Yue T.; Zhang X. Cooperative Effect in Receptor-Mediated Endocytosis of Multiple Nanoparticles. ACS Nano 2012, 6, 3196–3205. 10.1021/nn205125e. 22429100

[ref36] Gupta R.; Rai B. Effect of Size and Surface Charge of Gold Nanoparticles on Their Skin Permeability: A Molecular Dynamics Study. Sci. Rep. 2017, 7, 45292 10.1038/srep45292. 28349970 PMC5368607

[ref37] Gimondi S.; Vieira de Castro J.; Reis R. L.; Ferreira H.; Neves N. M. On the Size-Dependent Internalization of Sub-Hundred Polymeric Nanoparticles. Colloids Surf., B 2023, 225, 113245 10.1016/j.colsurfb.2023.113245. 36905835

[ref38] Bally F.; Garg D. K.; Serra C. A.; Hoarau Y.; Anton N.; Brochon C.; Parida D.; Vandamme T.; Hadziioannou G. Improved Size-Tunable Preparation of Polymeric Nanoparticles by Microfluidic Nanoprecipitation. Polymer 2012, 53, 5045–5051. 10.1016/j.polymer.2012.08.039.

[ref39] Popa M. L.; Preda M. D.; Neacşu I. A.; Grumezescu A. M.; Ginghină O. Traditional vs. Microfluidic Synthesis of ZnO Nanoparticles. Int. J. Mol. Sci. 2023, 24, 1875 10.3390/ijms24031875. 36768199 PMC9916368

[ref40] Fabozzi A.; Sala F. D.; di Gennaro M.; Barretta M.; Longobardo G.; Solimando N.; Pagliuca M.; Borzacchiello A. Design of Functional Nanoparticles by Microfluidic Platforms as Advanced Drug Delivery Systems for Cancer Therapy. Lab Chip 2023, 23, 1389–1409. 10.1039/D2LC00933A. 36647782

[ref41] Ho Y. T.; Adriani G.; Beyer S.; Nhan P.-T.; Kamm R. D.; Kah J. C. Y. A Facile Method to Probe the Vascular Permeability of Nanoparticles in Nanomedicine Applications. Sci. Rep. 2017, 7, 707 10.1038/s41598-017-00750-3. 28386096 PMC5429672

[ref42] Brown T. D.; Habibi N.; Wu D.; Lahann J.; Mitragotri S. Effect of Nanoparticle Composition, Size, Shape, and Stiffness on Penetration Across the Blood–Brain Barrier. ACS Biomater. Sci. Eng. 2020, 6, 4916–4928. 10.1021/acsbiomaterials.0c00743. 33455287

[ref43] Gimondi S.; Guimarães C. F.; Vieira S. F.; Gonçalves V. M. F.; Tiritan M. E.; Reis R. L.; Ferreira H.; Neves N. M. Microfluidic Mixing System for Precise PLGA-PEG Nanoparticles Size Control. Nanomedicine Nanotechnol. Biol. Med. 2022, 102482 10.1016/j.nano.2021.102482. 34748958

[ref44] Bouïs D.; Hospers G. A. P.; Meijer C.; Molema G.; Mulder N. H. Endothelium in Vitro: A Review of Human Vascular Endothelial Cell Lines for Blood Vessel-Related Research. Angiogenesis 2001, 40, 91–102. 10.1023/A:1012259529167. 11806248

[ref45] Ahn K.; Pan S.; Beningo K.; Hupe D. A Permanent Human Cell Line (EA.Hy926) Preserves the Characteristics of Endothelin Converting Enzyme from Primary Human Umbilical Vein Endothelial Cells. Life Sci. 1995, 56, 2331–2341. 10.1016/0024-3205(95)00227-W. 7791520

[ref46] Abdelgawad I. Y.; Agostinucci K.; Ismail S. G.; Grant M. K. O.; Zordoky B. N. EA.Hy926 Cells and HUVECs Share Similar Senescence Phenotypes but Respond Differently to the Senolytic Drug ABT-263. Cell 2022, 11, 1992 10.3390/cells11131992. PMC9266052 35805077

[ref47] Cho Y.-I.; Cho D. J. Hemorheology and Microvascular Disorders. Korean Circ. J. 2011, 41, 287 10.4070/kcj.2011.41.6.287. 21779279 PMC3132688

[ref48] Xia H. M.; Wan S. Y. M.; Shu C.; Chew Y. T. Chaotic Micromixers Using Two-Layer Crossing Channels to Exhibit Fast Mixing at Low Reynolds Numbers. Lab Chip 2005, 5, 748–755. 10.1039/B502031J. 15970968

[ref49] Aref H. The Development of Chaotic Advection. Phys. Fluids 2002, 14, 1315–1325. 10.1063/1.1458932.

[ref50] Aref H. Stirring by Chaotic Advection. J. Fluid Mech. 1984, 143, 1–21. 10.1017/S0022112084001233.

[ref51] Li J.; Kleinstreuer C. Microfluidics Analysis of Nanoparticle Mixing in a Microchannel System. Microfluid. Nanofluid. 2009, 6, 661–668. 10.1007/s10404-008-0341-1.

[ref52] Morikawa Y.; Tagami T.; Hoshikawa A.; Ozeki T. The Use of an Efficient Microfluidic Mixing System for Generating Stabilized Polymeric Nanoparticles for Controlled Drug Release. Biol. Pharm. Bull. 2018, 41, 899–907. 10.1248/bpb.b17-01036. 29863078

[ref53] Li W.; Chen Q.; Baby T.; Jin S.; Liu Y.; Yang G.; Zhao C.-X. Insight into Drug Encapsulation in Polymeric Nanoparticles Using Microfluidic Nanoprecipitation. Chem. Eng. Sci. 2021, 235, 116468 10.1016/j.ces.2021.116468.

[ref54] Suk J. S.; Xu Q.; Kim N.; Hanes J.; Ensign L. M. PEGylation as a Strategy for Improving Nanoparticle-Based Drug and Gene Delivery. Adv. Drug Delivery Rev. 2016, 99, 28–51. 10.1016/j.addr.2015.09.012. PMC4798869 26456916

[ref55] Libi S.; Calenic B.; Astete C. E.; Kumar C.; Sabliov C. M. Investigation on Hemolytic Effect of Poly(Lactic Co-Glycolic) Acid Nanoparticles Synthesized Using Continuous Flow and Batch Processes. Nanotechnol. Rev. 2017, 6, 209–220. 10.1515/ntrev-2016-0045.

[ref56] Bakhaidar R.; Green J.; Alfahad K.; Samanani S.; Moollan N.; O’Neill S.; Ramtoola Z. Effect of Size and Concentration of PLGA-PEG Nanoparticles on Activation and Aggregation of Washed Human Platelets. Pharmaceutics 2019, 11, 514 10.3390/pharmaceutics11100514. 31590303 PMC6835715

[ref57] Abbas Z.; Labbez C.; Nordholm S.; Ahlberg E. Size-Dependent Surface Charging of Nanoparticles. J. Phys. Chem. C 2008, 112, 5715–5723. 10.1021/jp709667u.

[ref58] Xiao K.; Li Y.; Luo J.; Lee J. S.; Xiao W.; Gonik A. M.; Agarwal R.; Lam K. S. The Effect of Surface Charge on in Vivo Biodistribution of PEG-Oligocholic Acid Based Micellar Nanoparticles. Biomaterials 2011, 32, 3435–3446. 10.1016/j.biomaterials.2011.01.021. 21295849 PMC3055170

[ref59] Sujai P. T.; Joseph M. M.; Saranya G.; Nair J. B.; Murali V. P.; Maiti K. K. Surface Charge Modulates the Internalization vs. Penetration of Gold Nanoparticles: Comprehensive Scrutiny on Monolayer Cancer Cells, Multicellular Spheroids and Solid Tumors by SERS Modality. Nanoscale 2020, 12, 6971–6975. 10.1039/D0NR00809E. 32202584

[ref60] Osaka T.; Nakanishi T.; Shanmugam S.; Takahama S.; Zhang H. Effect of Surface Charge of Magnetite Nanoparticles on Their Internalization into Breast Cancer and Umbilical Vein Endothelial Cells. Colloids Surf., B 2009, 71, 325–330. 10.1016/j.colsurfb.2009.03.004. 19361963

[ref61] Ye H.; Shen Z.; Yu L.; Wei M.; Li Y. Manipulating Nanoparticle Transport within Blood Flow through External Forces: An Exemplar of Mechanics in Nanomedicine. Philos. Trans. R. Soc., A 2018, 474, 20170845 10.1098/rspa.2017.0845. PMC5897762 29662344

[ref62] Bai Y.; An N.; Chen D.; Liu Y.; Liu C.; Yao H.; Wang C.; Song X.; Tian W. Facile Construction of Shape-Regulated β-Cyclodextrin-Based Supramolecular Self-Assemblies for Drug Delivery. Carbohydr. Polym. 2020, 231, 115714 10.1016/j.carbpol.2019.115714. 31888845

[ref63] Zhang F.; Aquino G. V.; Bruce E. D. A Quantitative and Non-Invasive Method for Nanoparticle Translocation and Toxicity Evaluation in a Human Airway Barrier Model. MethodsX 2020, 7, 100869 10.1016/j.mex.2020.100869. 32382518 PMC7199013

[ref64] Suttitheptumrong A.; Rawarak N.; Reamtong O.; Boonnak K.; Pattanakitsakul S.-N. Plectin Is Required for Trans-Endothelial Permeability: A Model of Plectin Dysfunction in Human Endothelial Cells After TNF-α Treatment and Dengue Virus Infection. Proteomics 2018, 18, e1800215 10.1002/pmic.201800215. 30365215

[ref65] Jeya Paul J.; Weigel C.; Müller T.; Heller R.; Spiegel S.; Gräler M. H. Inflammatory Conditions Disrupt Constitutive Endothelial Cell Barrier Stabilization by Alleviating Autonomous Secretion of Sphingosine 1-Phosphate. Cell 2020, 9, 928 10.3390/cells9040928. PMC7226983 32290092

[ref66] Samsonov M. V.; Khapchaev A. Y.; Vorotnikov A. V.; Vlasik T. N.; Yanushevskaya E. V.; Sidorova M. V.; Efremov E. E.; Lankin V. Z.; Shirinsky V. P. Impact of Atherosclerosis- and Diabetes-Related Dicarbonyls on Vascular Endothelial Permeability: A Comparative Assessment. Oxid. Med. Cell. Longevity 2017, 2017, 1625130 10.1155/2017/1625130. PMC5643129 29098058

[ref67] Gao H.; Zhang J.; Liu T.; Shi W. Rapamycin Prevents Endothelial Cell Migration by Inhibiting the Endothelial-to-Mesenchymal Transition and Matrix Metalloproteinase-2 and -9: An in Vitro Study. Mol. Vis. 2011, 17, 3406–3414. 22219636 PMC3247170

[ref68] Yoo J.-W.; Chambers E.; Mitragotri S. Factors That Control the Circulation Time of Nanoparticles in Blood: Challenges, Solutions and Future Prospects. Curr. Pharm. Des. 2010, 16, 2298–2307. 10.2174/138161210791920496. 20618151

[ref69] Fan W.; Peng H.; Yu Z.; Wang L.; He H.; Ma Y.; Qi J.; Lu Y.; Wu W. The Long-Circulating Effect of Pegylated Nanoparticles Revisited via Simultaneous Monitoring of Both the Drug Payloads and Nanocarriers. Acta Pharm. Sin. B 2022, 12, 2479–2493. 10.1016/j.apsb.2021.11.016. 35646531 PMC9136618

[ref70] Dal N.-J. K.; Kocere A.; Wohlmann J.; Van Herck S.; Bauer T. A.; Resseguier J.; Bagherifam S.; Hyldmo H.; Barz M.; De Geest B. G.; Fenaroli F. Zebrafish Embryos Allow Prediction of Nanoparticle Circulation Times in Mice and Facilitate Quantification of Nanoparticle–Cell Interactions. Small 2020, 16, 1906719 10.1002/smll.201906719. 31943784

[ref71] Lipka J.; Semmler-Behnke M.; Sperling R. A.; Wenk A.; Takenaka S.; Schleh C.; Kissel T.; Parak W. J.; Kreyling W. G. Biodistribution of PEG-Modified Gold Nanoparticles Following Intratracheal Instillation and Intravenous Injection. Biomaterials 2010, 31, 6574–6581. 10.1016/j.biomaterials.2010.05.009. 20542560

[ref72] Kaplan M.; Öztürk K.; Öztürk S. C.; Tavukçuoğlu E.; Esendağlı G.; Calis S. Effects of Particle Geometry for PLGA-Based Nanoparticles: Preparation and In Vitro/In Vivo Evaluation. Pharmaceutics 2023, 15, 175 10.3390/pharmaceutics15010175. 36678804 PMC9862984

[ref73] Da Silva-Candal A.; Brown T.; Krishnan V.; Lopez-Loureiro I.; Ávila-Gómez P.; Pusuluri A.; Pérez-Díaz A.; Correa-Paz C.; Hervella P.; Castillo J.; Mitragotri S.; Campos F. Shape Effect in Active Targeting of Nanoparticles to Inflamed Cerebral Endothelium under Static and Flow Conditions. J. Controlled Release 2019, 309, 94–105. 10.1016/j.jconrel.2019.07.026. 31330214

[ref74] Mazumdar S.; Chitkara D.; Mittal A. Exploration and Insights into the Cellular Internalization and Intracellular Fate of Amphiphilic Polymeric Nanocarriers. Acta Pharm. Sin. B 2021, 11, 903–924. 10.1016/j.apsb.2021.02.019. 33996406 PMC8105776

[ref75] Wu X.; Tang T.; Wei Y.; Cummins K. A.; Wood D. K.; Pang H.-B. Extracellular Vesicles Mediate the Intercellular Exchange of Nanoparticles. Adv. Sci. 2022, 9, 2102441 10.1002/advs.202102441. PMC8895114 35243822

[ref76] Yazdani S.; Jaldin-Fincati J. R.; Pereira R. V. S.; Klip A. Endothelial Cell Barriers: Transport of Molecules between Blood and Tissues. Traffic (Copenhagen, Denmark) 2019, 20, 390–403. 10.1111/tra.12645. 30950163

[ref77] Wu S. P.; Ringgaard S.; Oyre S.; Hansen M. S.; Rasmus S.; Pedersen E. M. Wall Shear Rates Differ between the Normal Carotid, Femoral, and Brachial Arteries: An in Vivo MRI Study. J. Magn. Reson. Imaging 2004, 19, 188–193. 10.1002/jmri.10441. 14745752

[ref78] Kang T.; Park C.; Lee B.-J. Investigation of Biomimetic Shear Stress on Cellular Uptake and Mechanism of Polystyrene Nanoparticles in Various Cancer Cell Lines. Arch. Pharmacal Res. 2016, 39, 1663–1670. 10.1007/s12272-016-0847-0. 27761800

[ref79] Khor S. Y.; Vu M. N.; Pilkington E. H.; Johnston A. P. R.; Whittaker M. R.; Quinn J. F.; Truong N. P.; Davis T. P. Elucidating the Influences of Size, Surface Chemistry, and Dynamic Flow on Cellular Association of Nanoparticles Made by Polymerization-Induced Self-Assembly. Small 2018, 14, 1801702 10.1002/smll.201801702. 30043521

[ref80] Samuel S. P.; Jain N.; O’Dowd F.; Paul T.; Kashanin D.; Gerard V. A.; Gun’ko Y. K.; Prina-Mello A.; Volkov Y. Multifactorial Determinants That Govern Nanoparticle Uptake by Human Endothelial Cells under Flow. Int. J. Nanomed. 2012, 7, 2943–2956. 10.2147/IJN.S30624. PMC3384367 22745555

[ref81] Satcher R.; Dewey C. F.; Hartwig J. H. Mechanical Remodeling of the Endothelial Surface and Actin Cytoskeleton Induced by Fluid Flow. MICROCIRCULATION 1997, 4, 439–453. 10.3109/10739689709146808. 9431512

[ref82] Tabouillot T.; Muddana H. S.; Butler P. J. Endothelial Cell Membrane Sensitivity to Shear Stress Is Lipid Domain Dependent. Cell. Mol. Bioeng. 2011, 4, 169–181. 10.1007/s12195-010-0136-9. 22247740 PMC3254098

[ref83] Qin X.; Zhang Y.; He Y.; Chen K.; Zhang Y.; Li P.; Jiang Y.; Li S.; Li T.; Yang H.; Wu C.; Zheng C.; Zhu J.; You F.; Liu Y. Shear Stress Triggered Circular Dorsal Ruffles Formation to Facilitate Cancer Cell Migration. Arch. Biochem. Biophys. 2021, 709, 108967 10.1016/j.abb.2021.108967. 34157295

[ref84] Press A. T.; Traeger A.; Pietsch C.; Mosig A.; Wagner M.; Clemens M. G.; Jbeily N.; Koch N.; Gottschaldt M.; Bézière N.; Ermolayev V.; Ntziachristos V.; Popp J.; Kessels M. M.; Qualmann B.; Schubert U. S.; Bauer M. Cell Type-Specific Delivery of Short Interfering RNAs by Dye-Functionalised Theranostic Nanoparticles. Nat. Commun. 2014, 5, 5565 10.1038/ncomms6565. 25470305 PMC4268698

[ref85] Rinkenauer A. C.; Press A. T.; Raasch M.; Pietsch C.; Schweizer S.; Schwörer S.; Rudolph K. L.; Mosig A.; Bauer M.; Traeger A.; Schubert U. S. Comparison of the Uptake of Methacrylate-Based Nanoparticles in Static and Dynamic in Vitro Systems as Well as in Vivo. J. Controlled Release 2015, 216, 158–168. 10.1016/j.jconrel.2015.08.008. 26277064

[ref86] Mahto S. K.; Yoon T. H.; Rhee S. W. A New Perspective on in Vitro Assessment Method for Evaluating Quantum Dot Toxicity by Using Microfluidics Technology. Biomicrofluidics 2010, 4, 034111 10.1063/1.3486610. 20957065 PMC2955720

[ref87] Yazdimamaghani M.; Barber Z. B.; Hadipour Moghaddam S. P.; Ghandehari H. Influence of Silica Nanoparticle Density and Flow Conditions on Sedimentation, Cell Uptake, and Cytotoxicity. Mol. Pharmaceutics 2018, 15, 2372–2383. 10.1021/acs.molpharmaceut.8b00213. 29719153

[ref88] Fede C.; Fortunati I.; Weber V.; Rossetto N.; Bertasi F.; Petrelli L.; Guidolin D.; Signorini R.; De Caro R.; Albertin G.; Ferrante C. Evaluation of Gold Nanoparticles Toxicity towards Human Endothelial Cells under Static and Flow Conditions. Microvasc. Res. 2015, 97, 147–155. 10.1016/j.mvr.2014.10.010. 25446009

[ref89] Sohn L. L.; Schwille P.; Hierlemann A.; Tay S.; Samitier J.; Fu J.; Loskill P. How Can Microfluidic and Microfabrication Approaches Make Experiments More Physiologically Relevant?. Cell Syst. 2020, 11, 209–211. 10.1016/j.cels.2020.07.003. 32888419 PMC7890516

[ref90] Koch E. V.; Ledwig V.; Bendas S.; Reichl S.; Dietzel A. Tissue Barrier-on-Chip: A Technology for Reproducible Practice in Drug Testing. Pharmaceutics 2022, 14, 1451 10.3390/pharmaceutics14071451. 35890346 PMC9323870

