# Supplementary material for: Size-Dependent Polymeric Nanoparticle Distribution in a Static versus Dynamic Microfluidic Blood Vessel Model: Implications for Nanoparticle-Based Drug Delivery
Source: ACS Appl Nano Mater. 2023 Apr 24;6(9):7364–74. doi: 10.1021/acsanm.3c00481 (PMC10189782; doi:10.1021/acsanm.3c00481)
Supplement: Supplementary file 1 — an3c00481_si_001.pdf [file an3c00481_si_001.pdf]

Supporting Information

Size-Dependent Polymeric Nanoparticle Distribution in a Static versus Dynamic Microfluidic Blood Vessel Model: Implications for Nanoparticle-Based Drug Delivery

Sara Gimondi[1,2], Helena Ferreira[1,2], Rui L. Reis[1,2], Neves M. Neves[1,2]* (nuno@i3bs.uminho.pt)

[1]3B's Research Group, I3Bs – Research Institute on Biomaterials, Biodegradables and Biomimetics, University of Minho, Headquarters of the European Institute of Excellence on Tissue Engineering and Regenerative Medicine, AvePark, Parque de Ciência e Tecnologia, Zona Industrial da Gandra, 4805-017 Barco, Guimarães, Portugal;

[2]ICVS/3B's–PT Government Associate Laboratory, Braga/Guimarães, Portugal;

Figure S1 and Table S1 report additional data regarding the characterization of the NPs after synthesis, namely the size distribution by intensity, volume, and number.

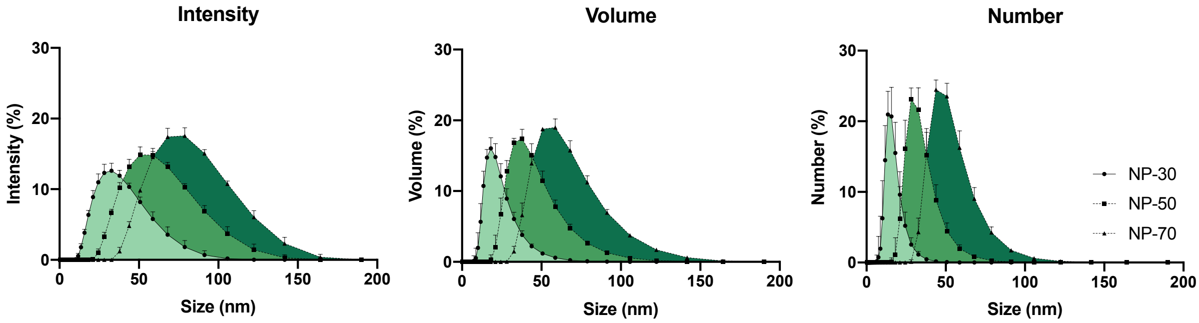

Figure S1 - NPs size characterization. *Size distribution by intensity, volume, and number expressed as percentage (%).*

Table S1 - *NPs intensity-, volume- and number-distribution size. Values are reported as average ± SD of three independent measurements.*

NP-FITC	Intensity (nm)	Volume (nm)	Number (nm)
30 nm	33.8 ± 2.0	21.8 ± 1.2	16.14 ± 1.6
50 nm	52.7 ± 1.3	42.2 ± 1.6	33.0 ± 2.0
70 nm	70.2 ± 1.2	62.9 ± 0.9	51.6 ± 1.8

In addition to the NPs stability evaluation along 1 month in static conditions, the integrity of NPs was assessed under shear conditions. The results are reported in Table S2.

Table S2 - NPs stability in dynamic conditions. *Intensity-, volume-, number-distribution size, PDI, ζ-potential, and fluorescence emission (FE) of NPs before (t=0 h) and after (t=4 h) their perfusion inside the microfluidic device. Values are reported as average ± SD of three independent measurements.*

Time	NPs	Intensity (nm)	PDI	ζ-potential (mV)	Volume (nm)	Number (nm)	FE
0h	30 nm	29.0 ± 1.5	0.13 ± 0.01	-19.1 ± 2.1	22.3 ± 0.8	17.1 ± 1.0	2905 ± 16
	50 nm	50.8 ± 1.7	0.11 ± 0.01	-22.0 ± 1.9	42.3 ± 1.6	33.3 ± 2.2	3538 ± 35
	70 nm	71.4 ± 1.3	0.07 ± 0.01	-25.4 ± 3.2	63.3 ± 0.9	52.4 ± 1.7	4178 ± 29
4h	30 nm	30.3 ± 1.8	0.15 ± 0.01	-18.9 ± 2.9	21.5 ± 1.4	15.4 ± 1.8	2900 ± 20
	50 nm	52.1 ± 1.6	0.11 ± 0.01	-20.3± 1.5	42.8 ± 1.4	33.5 ± 1.7	3516 ± 47
	70 nm	72.0 ± 1.4	0.08 ± 0.01	-25.1 ± 2.2	62.6 ± 1.0	50.7 ± 1.4	4170 ± 33

Figure S2 reports the brightfield images of the microfluidic BE-Doubleflow device with or without cells.

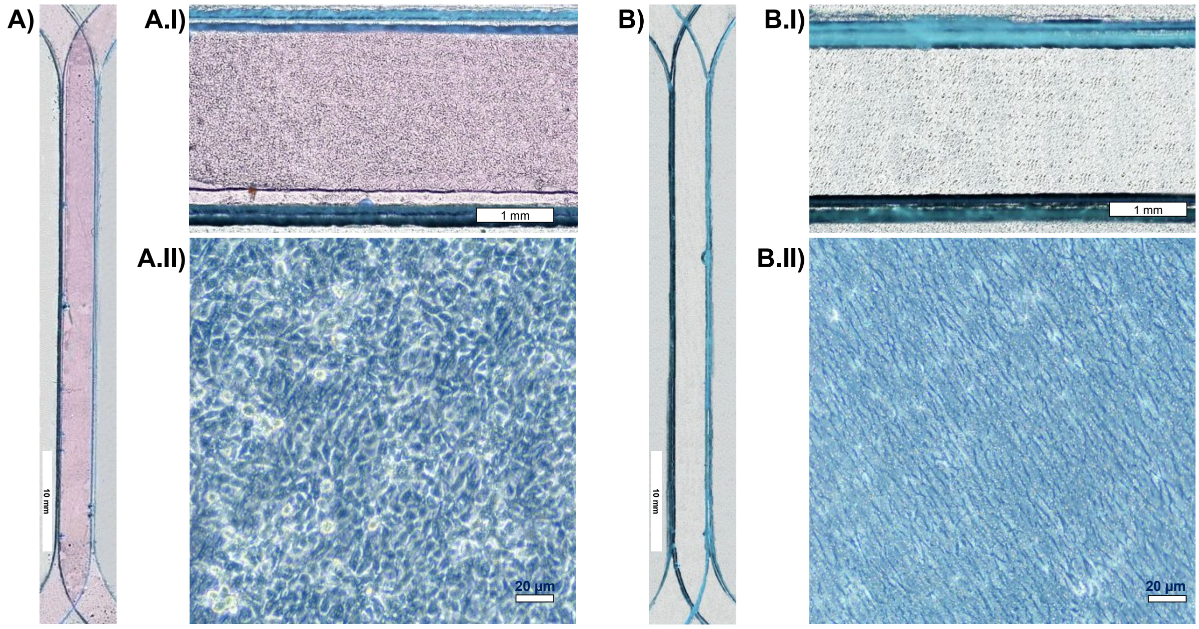

Figure S2 – Dynamic model. *Images of the whole BE-Doubleflow device in presence of cells at magnification 4x (A, scale bar 10 mm and A.I, scale bar 1 mm) and 20x (A.II, scale bar 20 µm) and without cells (blk) at magnification 4x (B, scale bar 10 mm and B.I, scale bar 1 mm) and 20x (B.II, scale bar 20 µm).*
